# Benthic Trophic Interactions in an Antarctic Shallow Water Ecosystem Affected by Recent Glacier Retreat

**DOI:** 10.1371/journal.pone.0141742

**Published:** 2015-11-11

**Authors:** Francesca Pasotti, Leonardo Ariel Saravia, Marleen De Troch, Maria Soledad Tarantelli, Ricardo Sahade, Ann Vanreusel

**Affiliations:** 1 Marine Biology Laboratory, Ghent University, Krijgslaan 281/S8, B-9000, Ghent, Belgium; 2 Institute of Sciences, National University of General Sarmiento, Juan María Gutierrez 1150, C.P.1613, Los Polvorines, Buenos Aires, Argentina; 3 Institute of Animal Diversity and Ecology, CONICET, Fac.Cs. E.F. y Nat. National University of Cordoba, Av. Vélez Sársfield 299, 5000, Córdoba, Argentina; Université du Québec à Rimouski, CANADA

## Abstract

The western Antarctic Peninsula is experiencing strong environmental changes as a consequence of ongoing regional warming. Glaciers in the area are retreating rapidly and increased sediment-laden meltwater runoff threatens the benthic biodiversity at shallow depths. We identified three sites with a distinct glacier-retreat related history and different levels of glacial influence in the inner part of Potter Cove (King George Island, South Shetland Islands), a fjord-like embayment impacted since the 1950s by a tidewater glacier retreat. We compared the soft sediment meio- and macrofauna isotopic niche widths (δ^13^C and δ^15^N stable isotope analysis) at the three sites to investigate possible glacier retreat-related influences on benthic trophic interactions. The isotopic niches were locally shaped by the different degrees of glacier retreat-related disturbance within the Cove. Wider isotopic niche widths were found at the site that has become ice-free most recently, and narrower niches at the older ice-free sites. At an intermediate state of glacier retreat-related disturbance (e.g. via ice-growler scouring) species with different strategies could settle. The site at the earliest stage of post-retreat development was characterized by an assemblage with lower trophic redundancy. Generally, the isotopic niche widths increased with increasing size spectra of organisms within the community, excepting the youngest assemblage, where the pioneer colonizer meiofauna size class displayed the highest isotopic niche width. Meiofauna at all sites generally occupied positions in the isotopic space that suggested a detrital-pool food source and/or the presence of predatory taxa. In general ice scour and glacial impact appeared to play a two-fold role within the Cove: i) either stimulating trophic diversity by allowing continuous re-colonization of meiofaunal species or, ii) over time driving the benthic assemblages into a more compact trophic structure with increased connectedness and resource recycling.

## Introduction

The West Antarctic Peninsula (WAP) has been one of the Earth’s most rapidly warming regions over recent decades [1–5], with obvious consequences such as ice-sheet thinning [6], widespread retreat of glacier fronts [7], retreat and collapse of ice-shelves [8,9] and acceleration of snow melting [10]). The warmer air temperatures lead to rapid local increases, during the summer months, in glacial meltwater discharge and enhanced snow and permafrost melting [11]. In the coastal marine environment, these two processes are responsible for changes in water column turbidity and stratification [11,12], local increases in inorganic sedimentation [13], the appearance of newly available ice-free substrata [14–16], and changes in the frequency and scale of ice disturbance [17].

Disturbance is a key factor in structuring benthic communities worldwide [18–21]. In the Antarctic, one of the major structuring forces involved in shaping the shallow benthos is ice scouring [21–29], including the formation of anchor ice [30–32]. Other factors such as sedimentation have been seen to affect Arctic meio- and macrobenthos distribution [33–36], Antarctic soft coral survival (after a slumping event, [29]), and the physiology and survival of Antarctic key species such as the bivalve *Laternula elliptica*, ascidians and the sea-pen *Malacobelemnon daytoni* [35,37]. The potential crossing of sedimentation and meltwater input thresholds as climate change advances in the West Antarctic has been identified as a considerable threat to fjord biodiversity hotspots [38]. However a positive feature of this process is the availability of newly ice-free substrata that may stimulate new colonization and initiate succession processes, as recently documented for Antarctic marine [39,40] and terrestrial ecosystems [15,16,41,42]. Although not unique in the history of the WAP [5], the present rate of environmental changes can be considered to be a greater disturbance to shallow marine communities than any previously identified. Such changes, therefore, constitute potential stress factors that may alter community composition and species interactions.

Studies on the influence of glacier-related effects on benthic biota have, so far, focused on the investigation of individual species responses [33–36], or on the description of benthic community structure [43–46]. Recently, important shifts in macro-epibenthic community structure were related to changes in glacial influence at the site targeted by the current study, Potter Cove (King George Island, South Shetland Islands) [43]. These initial structural observations at present lack information on the functional responses of benthic communities to the changing environment. Although a number of food web studies describe trophic relationships in the Antarctic benthos [47–51], no study has yet attempted to link glacier retreat at the local scale and related environmental conditions to changes in benthic trophic interactions.

In this investigation, we investigated in detail the trophic structure of three benthic assemblages at three sites considered to represent a time-integrated snapshot of glacier retreat effects on shallow water benthic trophic organization. Based on observations at three shallow stations (15 m depth) in Potter Cove, we recently showed how community structure of the benthos (microbiota, meio- and macrofauna) varied along a virtual line of “age since glacier retreat” as represented by distance from the current glacier front [44]. Since the 1950’s the glacier has been actively retreating, exposing the previously underlying sediments to open water dynamics, where seasonal melt water discharge affects the shallow benthic habitats. Moreover, ice growlers (small icebergs) calving from the glacier impact with variable frequency the seabed of the three sites. Following the observations of Rückamp et al. [52], Creek was identified as the first of the studied locations to become ice-free (in the early 1950s). Due to its location, the conformation of the glacier and the clock-wise current system of Potter Cove [53], the site is the one which has experienced the consequences the glacier’s retreat the longest. Next, Faro station became ice-free over about 7–8 years in the 1990s. During this time period the retreating glacier front was very close to this newly ice-free island, and the local benthic assemblage likely suffered from inorganic load, drop stones and ice growler impact for a prolonged period. However, today, the Faro site seems to be the least affected by growler impact [34] and experiences the lowest sediment accumulation rates [44] although with high bed shear stress [54]. The most recent area to become ice-free (2003–2006) is that surrounding the Isla D site. Isla D station records the highest sediment accumulation rates of the three study sites, and is likely to continue to be strongly affected by ice impact as reflected in the patchy distribution of the endobenthic assemblage [44]. Focusing on these known sites within Potter Cove, in this study we investigated whether the local glacier retreat had detectable effects on trophic interactions in the benthic assemblage, through applying techniques to describe their isotopic niche distribution in order to identify the positions of different organisms (or size classes) within the food web. To gain this improved insight into trophic links in these Potter Cove communities, we analysed dual stable isotopic signatures (δ^13^C and δ^15^N) of two benthic size classes (meio- and macrofauna). Our hypothesis are that i) the three sites assemblages display different overall food web structures and that ii) the investigated size classes show different responses to the site-specific glacier-retreat history.

## Material and Methods

This investigation was carried out with approval of the governing body of Argentina’s Antarctic activities, the Dirección Nacional del Antártico. No special permissions were necessary for the activities carried out within this work since the investigated sites did not comprise any Specially Protected Area. The study did not involve endangered or protected species.

### Study site

**Fig 1:**
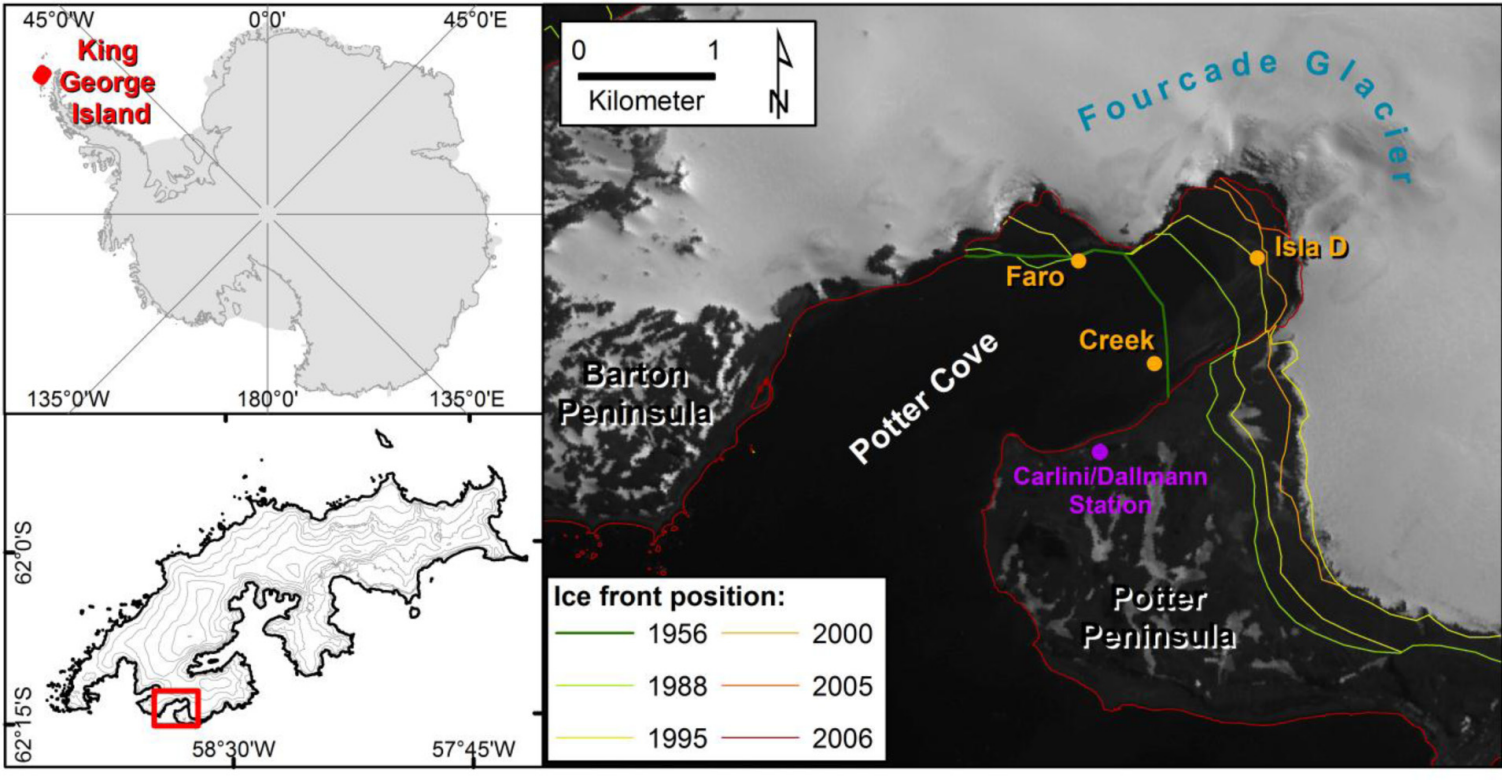
Map of Potter Cove. Potter Cove location within the Antarctic (from Pasotti et al., 2014b). The history of retreat of the Fourcade glacier is indicated by the ice front position lines for each different years.

Potter Cove is a small fjord-like bay the south coast of King George Island (South Shetland Islands, Fig 1). The cove is characterized by the recent retreat of the Fourcade glacier [52]. Additional freshwater input originates from seasonal meltwater discharge as a consequence of permafrost and snow melting processes. The three shallow water (15 m) stations included in the present study (see Fig 1) are located in the inner part of the cove and are mainly characterized by soft sediment [44,54]. Isla D station (62° 13' 32.6" S, 58° 38' 32" W) is the most recently ice-free area (2003–2006), and is situated closest to the glacier front. Faro station (62° 13' 32.6" S, 58° 40' 03.7"W) lies near the northern shore of the cove and became ice-free between 1988 and 1995. Creek station (62° 13‘57.3" S, 58° 39’ 25.9" W) is located adjacent to a seasonal meltwater river (“Potter Creek”) and has been ice-free since the early 1950s.

### Sampling and stable isotope analyses

Samples for stable isotopes analysis were obtained during two campaigns in February/March 2011 and March 2012. The majority of potential food sources were sampled during the first campaign, whereas microphytobenthos could only be collected during the second season. Meiofauna and macrofauna were mostly collected during the first campaign. From the second sampling campaign, two cumacean species collected at Isla D and polychaetes (suborder Terebellida, family Ampharetidae) were added to the dataset. The majority of collected organisms were stored in formaldehyde, with the remainder being stored frozen (-20°C) prior to stable isotope analyses. In view of the depleting effect of formaldehyde on background stable isotopic carbon signatures [55–57], we applied a correction factor of +2‰ to the δ^13^C values [57] of those invertebrate samples that were stored in formalin, as these were processed within 6 (macrofauna, buffered formalin, 8%) to 12 (meiofauna, buffered formalin, 4%) months of sampling. No lipid extraction or mathematical normalization was applied to the samples. However, the known deleterious effect of formalin fixation on lipid preservation [58] and the good fitting of the formalin-corrected carbon data in relation to the putative sources when compared to other known values from literature suggest that the effect of lipids in the overall data interpretation from our samples can be considered minimal. As the main focus of this study is a comparative analysis of the three sites’ food webs, and not a detailed investigation of trophic links within these webs, carbon data are presented as secondary and supplementary information. The dual isotopic composition (carbon and nitrogen) of the samples was analyzed using a PDZ Europa ANCA-GSL elemental analyzer 230 interfaced to a PDZ Europa 20–20 isotope ratio mass spectrometer (Sercon Ltd., Cheshire, UK; UC Davis Stable Isotope Facility, http://stableisotopefacility.ucdavis.edu/). All isotope data were expressed in standard δ notation (measured as ‰), comparing the ratio of the heavy/light isotope to standard reference materials (Pee Dee belemnite for carbon and atmospheric N_2_ for nitrogen). All organisms were washed with milliQ water (when applicable) and dried overnight at 60°C. After drying, the material was ground to a fine powder using a mortar and placed in Al (for dual - δ^13^C and δ^15^ N–or solely δ^15^N analysis) or Ag (for δ^13^C analysis when acidification was required) capsules (6.5–8 mm). In the latter case, all carbonates were removed prior to the δ^13^C analysis through acidification with “drop by drop” addition of HCl of specific concentrations (details below). All capsules were dried, pinched closed and kept dry until further analysis.

#### Suspended Particulate Matter (SPM)

Water column SPM data were obtained from water samples taken with Niskin bottles from 0 to 30 m depth at the inner and the outer cove during the austral summers 2007–2008 and 2008–2009. The water samples were filtered with GF-F glass fiber filters (47 mm diameter, 0.7 μm pore size), treated drop by drop with 1 M HCl and rinsed with distilled water to eliminate carbonates. Lipids were extracted in Chloroform-methanol (2:1 vol:vol). The isotopic analysis was performed in a mass spectrometer (IRMS) (Thermo Finnigan Delta XP Plus) connected by a Thermo Finnigan Conflo III to an elemental analyzer (Thermo Flash EA 1112).

#### Phytoplankton and zooplankton

To obtain zooplankton and phytoplankton samples, seawater was filtered during horizontal tows (0–15 m, 15 min) with 220 μm and 55 μm mesh nets at each study site within the cove. However these were not considered as site-specific samples given the short distances between the stations and the current system within theCove. Each sample obtained was filtered on pre-combusted (500°C for 2h) GF/F glass fiber filters (47 mm diameter, 0.7 μm pore size). Filters were then stored frozen (-20°C) until further analysis. During the processing of microalgal material, each filter was examined under a dissection microscope and any zooplankton present were hand-picked and removed from the filter. The microalgae were then gently scooped from the filter with a sterile spoon-tipped needle and placed into the capsules. For zooplankton, only calanoid copepods and one large individual mysid provided sufficient biomass for dual stable isotope analysis.

#### Sediment

Sediment samples were obtained via SCUBA diving, using perspex push cores (5.6 cm inner diameter) at each site (2 replicates per site). One centimeter slices were cut from the surface down to 5 cm depth of sediment and placed into Petri dishes (diameter 90 mm). Samples were then stored frozen (-20°C) prior to further analysis. From each layer of each core, aliquots of sediment were taken for δ^13^C and δ^15^N stable isotope analysis. Sediment aliquots were acidified (in Ag capsules) with increasing concentrations of HCl (0.25 N, 1 N, 2 N) in order to avoid excessive bubbling and loss of sediment.

#### Microphytobenthos

Sediment with a brownish microphytobenthic layer was sampled via SCUBA diving using perspex push cores (5.6 cm inner diameter). Immediately after sampling, the top 1 cm of sediment was separated and stored in a Petri dish placed into a tray filled with ice in order to prevent pigment degradation. In the laboratory, microalgae were extracted from the sediment by placing lens tissue on its surface under an artificial light. Glass cover slips placed on top of the lens tissue were used to collect the microalgae migrating towards the light and adhering to the cover glass. The microalgal biofilm was then scraped off the slide, collected with pre-filtered seawater and filtered on a GF/F (pre-combusted 550°C) filter, and stored frozen (-20°C) until analysis. At the time of preparation for the dual stable isotope analysis, the filter was placed under a dissecting microscope and the visible microalgal mat was carefully scooped with a spoon-tip needle from the surface of the filter.

#### Macroalgae

Specimens of seven representative macroalgae species were hand-collected by SCUBA divers on the northern rocky shore of the cove, where macroalgae are most abundant [14]. The specimens were washed repeatedly with milliQ water and dried at 60°C for 48 h. The dried material was stored in Petri dishes and kept dry until further processing. During the processing, small sections of the dried algae were homogenized with a mortar and destined to the analysis.

#### Meiofauna

Sediment cores (5.6 cm inner diameter) were collected at each station by SCUBA diving, sliced as described in sub-section “Sediment”, and stored in 4% formaldehyde (buffered with pre-filtered sea water). Initial dual stable isotope analyses of nematodes, copepods and cumaceans from the formalin (4%) samples did not provide reliable dual stable isotope values and were therefore excluded from the analysis; those of polychaetes and amphipods were therefore used during these analyses. An extra replicate was therefore processed to gather material for nematodes, copepods and cumaceans. We sacrificed one replicate of the samples collected for sediment analysis (0–1 cm layer, stored at -20°C). Meiofaunal extraction followed standard procedures including centrifugation with LUDOX HS40, and sieving over 1000 and 32 μm sieves [59,60]. Extraction with LUDOX is known not to influence the natural stable isotopic signatures of metazoans [61]. Meiofauna were identified at higher taxon level following [62] and the most abundant groups (nematodes, copepods, cumaceans, polychaetes and amphipods) were separated for stable isotope analysis. For nematodes, 600 individuals were picked at random, washed repeatedly in milliQ water and filtered on pre-combusted (500°C) GF/F glass fiber filters (47 mm diameter, 0.7 μm pore size).

Harpacticoid copepods (40 individuals) were sorted into two morphotypes (MT) (e.g. belonging to different families/genera): MT1 without any epibiotic organisms; MT2 carried epibiotic ciliates on the exoskeleton. Cumaceans and polychaetes were grouped, when possible, at the family/species level. Cumaceans were acidified with 0.25 N HCl prior to further δ^13^C analysis.

#### Macrofauna

Macrofauna were sorted into two size classes. Endo/epibenthic organisms smaller than 1 cm were classified as “small macrofauna” (e.g. Cirratulidae polychaetes, amphipods etc.), and those larger than 1 cm (e.g. *Yoldia eightsi*, *Malacobelemnon daytoni*) were classified as “large macrofauna”.

In order to collect the “small” endobenthic and some of the “large” macrofauna (e.g. *Y*. *eigthsi*, *M*. *daytoni*, *Barrukia cristata*, *Aglaophamus trissophyllus*), sediment samples were obtained at each site by means of a Van Veen grab operated from a zodiac. The sediment was washed over a 1 mm mesh sieve and the animals were sorted alive, before being stored in 8% formaldehyde (buffered in pre-filtered seawater). At the time of sample preparation, when possible, polychaetes were grouped to family/species level or morphotypes, and amphipods and cumaceans were sorted to family or species level. Small crustaceans were treated, prior to drying, with 2N HCl to remove carbonates. For small taxa, several individuals were used for isotopic analysis. Larger molluscs (e.g. *Y*. *eightsi*) were removed from their shells and muscular foot tissue from individual organisms was collected. The internal calcareous rod of *M*. *daytoni* was removed from the colony soft body tissue and part of the colonial organism was dissected and carefully rinsed with milliQ water. Samples were acidified with 2N HCl. Given their previously recorded importance at this study site [22,44,63], selected “large” macrofauna were hand-collected by divers in the vicinity of the study sites, including the isopod *Paraserolis polita* and the ascidians *Molgula pedunculata*, *Cnemidocarpa verrucosa* and *Corella eumyota*. Upon collection these organisms were left overnight in pre-filtered seawater in order to allow gut clearance. The crustacean was frozen in liquid N_2_ and stored at -20°C until processing and analysis, whereas the ascidians were stored in formalin (8% final concentration). The muscle tissue from *P*. *polita* was removed using a sterilized bistoury. For the processing of the ascidians, the tunic of each individual ascidian was carefully dissected and rinsed in milliQ water. An epiphytic colonial ascidian was present on the outer tunic of one specimen of *C*. *eumyota*. This was carefully removed and prepared separately for stable isotope analysis (samples were acidified in 0.25 N HCl prior to δ^13^C analysis).

### Data analysis

We identified a total of 12 trophic groups being relevant for the benthic interactions under study. Potential food sources were classified into 6 groups: Suspended Particulate Matter (SPM), phytoplankton 55 μm, phytoplankton 200 μm, macroalgae, microphytobenthos (MPB) and sediment. Consumer taxa were classified into the feeding groups of zooplanktonic grazer, filter/suspension feeder, deposit feeder/omnivore, bearing epibiont, scavenger/omnivore or predator/omnivore, based on life traits reported in available literature (see Table 1).

**Table 1:** Stable isotope values.

Taxon		*N(R)*	δ^13^C	δ^15^N	Bayesian size grouping	Trophic group	References for trophic group
*** ***	Suspended Particulate Matter s	*22*	-26.08 ± 1.17	0.45 ± 1.17	NA	SPM	
***Algae***							
**Macroalgae**	Macroalgae	22	-25.03 ± 5.45	3.08 ± 0.85	NA	Macroalgae	
**Microphytobenthos**	Microphytobenthos	1(2)	-13.15 ± 0.35	4.90 ± 0.14	NA	MPB	
**Phytoplankton**	200 μm mesh	-5	-25.31 ± 1.47	4.33 ± 0.54	NA	Phytoplankton 200 μm	
	50 μm mesh	-3	-23,41 ± 0.85	4.69 ± 0.34	NA	Phytoplankton 55 μm	
**Sediment POM**	Sediment (Faro) 0–5 cm	10(2)	-20.81 ± 1.21	3.26 ± 0.77	NA	Sediment	
	Sediment (Faro) 0–1 cm	1(2)	-18.72 ± 0.56	3.35 ± 0.19	NA	Sediment	
	Sediment (Isla D) 0–5 cm	10	-18.84 ± 1.8	3.25 ± 0.89	NA	Sediment	
	Sediment (Isla D) 0–1 cm	1(2)	-17.84 ± 1.06	3.86 ± 0.67	NA	Sediment	
	Sediment (Creek) 0–5 cm	10	-22.54 ± 1.09	4.01 ± 0.68	NA	Sediment	
	Sediment (Creek) 0–1 cm	1(2)	-21.53 ± 1.6	4.55 ± 1.00	NA	Sediment	
***Cnidaria***							
**Pennatulacea**	*Malacobelemnon daytoni* (F)	1(3)	-22.64 ± 0.3	7.14 ± 0.22	Large Macrofauna	Filter/suspension feeder	[68,69]
***Tunicata***							
**Ascidiacea**	*Molgula pedunculata* (I)	1(2)	-23.57 ± 0.01	4.69 ± 0.00	Large Macrofauna	Filter/suspension feeder	[70,77,78]
	*Corella eumyota* (I)	1(3)	-23.99 ± 0.1	4.58 ± 0.08	Large Macrofauna	Filter/suspension feeder	“
	*Cnemidocarpa verrucosa* (I)	1(2)	-24.24 ± 0.23	5.83 ± 0.67	Large Macrofauna	Filter/suspension feeder	“
	Ascidian (I)	1	-25.48	3.52	Large Macrofauna	Filter/suspension feeder	[79]
**Crustacea**							
**Mysida**	Mysid	1	-21.36	6.1	NA	Zooplankton	[79,80]
**Tanaidacea**	Nototanaid MT1 (F)	7(2)	-20.92 ± 1.96	4.90 ± 0.66	Small Macrofauna	deposit feeder/omnivore	[81]
** **	Nototanaid MT2 (F)	8(3)	-15.48 ± 0.86	4.80 ± 0.20	Small Macrofauna	deposit feeder/omnivore	“
**Cumacea**	Eudorella sp. (F)	5(2)	-17.05 ± 0.07	7.65 ± 0.07	Small Macrofauna	deposit feeder/omnivore	“
** **	Eudorella sp. (I)	5(2)	-16.45 ± 1.06	7.05 ± 0.21	Small Macrofauna	deposit feeder/omnivore	“
** **	Eudorella sp. (C)	5(2)	-19.45 ± 0.84	7.05 ± 0.21	Small Macrofauna	deposit feeder/omnivore	“
** **	Dyastilis sp. (I)	5(2)	-21.8 ± 0.14	5.70 ± 0.00	Small Macrofauna	deposit feeder/omnivore	“
** **	Eudorella sp. (F)	8(2)	-16.63 ± 0.01	13.75 ± 0.6	Meiofauna	deposit feeder/omnivore	“
** **	Eudorella sp. (C)	8(1)	-14.07	10.07	Meiofauna	deposit feeder/omnivore	“
**Copepoda**	Calanoids copepods	3(7)	-24.19 ± 1.1	6.77 ± 0.38	NA	Zooplankton	
** **	Harpacticoids MT2 (I)	40(1)	-34.9	-2.6	Meiofauna	bearing ectosymbiont	[82]
** **	Harpacticoids MT1 (I)	40(1)	-20.05	10.34	Meiofauna	deposit feeder/omnivore	[83–85]
**Isopoda**	Paraserolis polita (F)	1(2)	-16.65 ± 1.06	12.1 ± 0.56	Large Macrofauna	predator/omnivore	[86]
**Amphipoda**	Phoxocephalidae (C)	3(5)	-16.2 ± 0.31	11.33 ± 0.27	Small Macrofauna	scavenger/omnivore	[87,88]
** **	Phoxocephalidae (F)	3(2)	-14.38 ± 0.75	7.83 ± 0.04	Small Macrofauna	scavenger/omnivore	“
** **	Amphipod MT1 (F)	1(3)	-17.61 ± 0.00	10.59 ± 0.01	Small Macrofauna	scavenger/omnivore	”
** **	Amphipodes (I)	5(2)	-19.71 ± 1.60	8.99 ± 0.70	Meiofauna	scavenger/omnivore	“
***Mollusca***							
**Bivalvia**	*Yoldia eightsi* (F)	1(3)	-11.81 ± 0.84	8.75 ± 0.42	Large Macrofauna	deposit feeder/omnivore	[67]
** **	*Yoldia eightsi* (C)	1(3)	-11.76 ± 0.81	8.12 ± 0.27	Large Macrofauna	deposit feeder/omnivore	“
***Nematoda***	Nematodes (F)	600(1)	-17.99	8.6	Meiofauna	deposit feeder/omnivore	[89]
	Nematodes (I)	600(1)	-16.5	8.25	Meiofauna	deposit feeder/omnivore	“
							“
	Nematodes (C)	600(1)	-18.5	10.1	Meiofauna	deposit feeder/omnivore	
***Polychaeta***							
Nephtydae	*Aglaophamus trissophyllus* (C+I)	1(3)	-14.27 ± 0.08	12.58 ± 0.07	Large Macrofauna	predator/omnivore	[90]
Polynoidae	*Barrukia cristata* (C+I)	1(3)	-16.96 ± 0.17	11.74 ± 0.06	Large Macrofauna	predator/omnivore	”
	Cerratulidae (F)	5(6)	-18.06 ± 0.45	8.35 ± 0.30	Small Macrofauna	deposit feeder/omnivore	“
	Cerratulidae (C)	5(3)	-14.12 ± 0.5	7.69 ± 0.18	Small Macrofauna	deposit feeder/omnivore	“
	Spionidae (F)	1(2)	-18.27 ± 0.27	8.64 ± 0.34	Small Macrofauna	deposit feeder/omnivore	“
	Opheliidae (F)	10(2)	-19.35 ± 0.42	11.52 ± 1.42	Small Macrofauna	deposit feeder/omnivore	“
	Capitellidae (F)	10(1)	-15.49	12.62	Small Macrofauna	deposit feeder/omnivore	“
	Capitellidae (C)	5(2)	-17.09 ± 0.58	9.85 ± 0.46	Small Macrofauna	deposit feeder/omnivore	“
	Maldanidae MT1 (F)	1(3)	-15.89 ± 0.06	10.34 ± 0.7	Small Macrofauna	deposit feeder/omnivore	“
	Maldanidae MT2 (F)	1(3)	-19.68 ± 0.14	9.64 ± 0.35	Small Macrofauna	deposit feeder/omnivore	“
	Capitellidae (F)	5(3)	-19.01 ± 0.10	12.49 ± 0.3	Meiofauna	deposit feeder/omnivore	“
	Cirratulidae MT1 (C)	5(3)	-18.30 ± 0.03	7.58 ± 0.04	Meiofauna	deposit feeder/omnivore	“
	Cirratulidae MT2 (C)	10(1)	-18.15 ± 0.57	12.36 ± 0.54	Meiofauna	deposit feeder/omnivore	“
	Orbiniidae (F)	5(3)	-19.34 ± 0.42	11.51 ± 1.41	Meiofauna	deposit feeder/omnivore	“
	Cirratulidae (F)	5(3)	-18.28 ± 0.03	11.26 ± 0.24	Meiofauna	deposit feeder/omnivore	“
	Ampharetidae (I)	1(1)	-15.90 ± 0.84	8.1 ± 1.14	Small Macrofauna	deposit feeder/omnivore	“
***Priapulida***	*Priapulus sp*.(F)	1(3)	-17.99 ± 0.44	10.7 ± 0.49	Small Macrofauna	predator/omnivore	[91,92]

Stable isotopes values (average ± standard deviation) of food sources and consumers. N(R) = number of individuals (per number of replicas); Bayesian modeling grouping = size class grouping used in the SIBER program. Where applicable taxa have the specification of the site: Creek (C), Faro (F), Isla D (I). The symbol “indicates that the citation is the same as above. NA = not available.

In order to allow a description of the community in terms of trophic levels (TL), we identified baseline estimates of trophic position following [64,65] and based on both our data observations and published background information on species life traits. Food sources showed a rather wide range of δ^15^N values, overlapping with values obtained from consumers. We identified therefore the range of the first level of consumers (TL2) of our food web based on available knowledge of the feeding habits and life history traits of the studied filter feeders and the long-lived deposit/suspension feeder *Y*. *eightsi*. The protobranch bivalve mollusc *Y*. *eightsi* represents an important component in terms of biomass at our sites [44]. The organisms used during our investigation were between 2–3 cm in length and hence likely older than 50 y [66]. *Y*. *eightsi* is known to be both a deposit feeder (on mud) and a suspension feeder (on diatoms in the ventilator stream) [67], but in our analysis it was included in the deposit feeder/omnivore category owing to its sediment-related functional characteristics. Based on carbon signature data, indicating an influence of microphytobenthos in its diet, we included *Y*. *eightsi* in the second trophic level (TL2) or first consumer category. Sea pens are known to be passive suspension-feeders on phytoplankton [68] and potentially very small zooplankton [69]. Ascidians in Potter Cove have been found to contain macroalgal fragments in their gut [70], besides potentially feeding on phytoplankton and other particulate organic matter present in the water column. We considered the benthic-pelagic compartment as part of a single food web in the light of the frequent re-suspension events in PC shallow waters [12,44], which permit suspension and filter feeders potentially to feed on both compartments at the same time, given their non-strict feeding selectivity. We decided to separate the next trophic (TL3-TL5) levels using intermediate δ^15^N trophic fractionation step derived from the work of McCutchan et al. [64] invertebrate-based and plant-based fractionation steps of 1.8‰, in place of the standard δ^15^N trophic fractionation of 3‰ [65,71].

We identified the trophic positions based on this information and the following formula: [65]

$$TP=\left[\lambda +(\delta^{15}N_{consumer}\;-\;\delta^{15}N_{base})\right]/\;\Delta_{n}$$

Where TP = trophic position of a particular species/group, λ is the trophic position of the chosen organism used for the estimation of δ^15^N_base_ (e.g., λ = 2 for primary consumers, 3 for secondary consumers), δ^15^N _consumer_ is measured directly, and Δ_n_ is the trophic enrichment (Δ^15^N, ‰) per level.

By means of Standard Ellipse Areas (SEAs, [72,73]) and Layman’s metrics [73], we compared the three shallow benthic assemblage isotopic niches in relation to the ongoing glacier retreat. In order to compare the benthic community isotopic niches at the three sites (“by site”) or between the three size classes at each site (“size by site”), we used Standard Ellipse Areas (SEA; expressed in ‰^2^; [75]). SEAs are comparable to the univariate standard deviation (SD) and contain about 40% of the variability of the dataset [74]. They are also more appropriate for unbalanced datasets and allow comparisons between communities with different numbers of taxa/groups [72]. A Bayesian approach was used to represent the estimated (posterior) distribution of SEAs taking into account uncertainty derived from the sampling process (SEA). We also calculated a point estimator of SEA corrected for small sample size (SEA_c_) [72]. Using SEA_c_ we calculated the overlaps between sites, identified by the areas in which ellipses were superimposed. All analyses were completed using the SIBER package (Stable Isotope Bayesian Ellipses in R; [72]) within the SIAR package in R. The R scripts used for the analysis are available at http://dx.doi.org/10.6084/m9.figshare.1094784. We tested normality with the multivariate Shapiro-Wilk test and graphically with quantile-quantile plots in R. One of the three sites did not meet the normality assumption but, since each of the datasets has sample size of n ≥ 30, SEA_c_ is robust to this violation [75]. In “size by site” analysis three of nine data distributions did not follow normality and the sample size was < 30 (n ~ 10), hence we could not use SEA_c_ to compare the overlaps. We tested the Bayesian model using a simple graphical method: we plotted together the single SEA_c_ and the SEA_b_ distribution. The metric calculated from the data (as single point SEA_c_) must lie inside the 95% credible intervals calculated from the Bayesian analysis [76].

In addition, we compared the three sites in terms of trophic structure using the community-wide metrics of Layman et al. [73]. These metrics were suited for comparison of the three sites in this study since the food source δ^13^C ranges were the same [72]. The same Bayesian approach was used to incorporate uncertainty and allow comparison of posterior probabilities. We calculated five metrics using groups defined as size classes (Table 1): (i) δ^13^C range (CR), indicative of niche diversification; (ii) δ^15^N range (NR), indicative of trophic length; (iii) mean distance from centroid (CD), indicative of the average trophic diversity; (iv) mean nearest neighbor distance (MNND), or a measure of the density of species packing; and (v) standard deviation of mean nearest neighbor distance (SDNND), or evenness of species packing. Together, the last two metrics are indicative of trophic redundancy: small MNND indicates higher trophic redundancy, where more species perform the same trophic function, and lower SDNND indicates more evenly distributed species with more species having similar ecological traits.

For the analysis we ran the SIBER analysis on two sets of data: i) the primary analysis based on the complete dataset (*“complete dataset”*), where we merged all the available information and samples from the three sites, and ii) the analysis without “ambiguous” data excluding possible outliers or species not found during the qualitative sampling for stable isotope analysis but known to be present at the study sites [44]. From this “*reduced dataset”* we excluded from Isla D i) the harpacticoid copepod MT2 since this species was as an outlier (very depleted δ^13^C and δ^15^N values) and ii) the two polychaete species *Barrukia cristata* (Polynoidae) and *Aglaophamus trissophyllus* (Nepthydae), since they were found during the qualitative sampling only at Creek station. Nevertheless, since a previous investigation [44] reported that these two polychaete species were an important component of the Isla D benthic community in summer 2011, and in light of the metabolic peculiarity of the very depleted values of the copepod MT2, we included these organisms in the”*complete dataset”* analysis.

## Results

Results are expressed as mean±SD. In Table 1 a list with the average δ^13^C and δ^15^N values of all the samples is presented. In the Supporting Information (S) the results from the “*reduced dataset*” analysis are given in S1–S3 Tables. The complete set of figures from the “*reduced dataset*” analysis are given in S1–S4 Figs, whereas a plot with the food source and consumer δ^13^C values is given as extra information in S5 Fig.

### Stable isotope signatures

**Fig 2:**
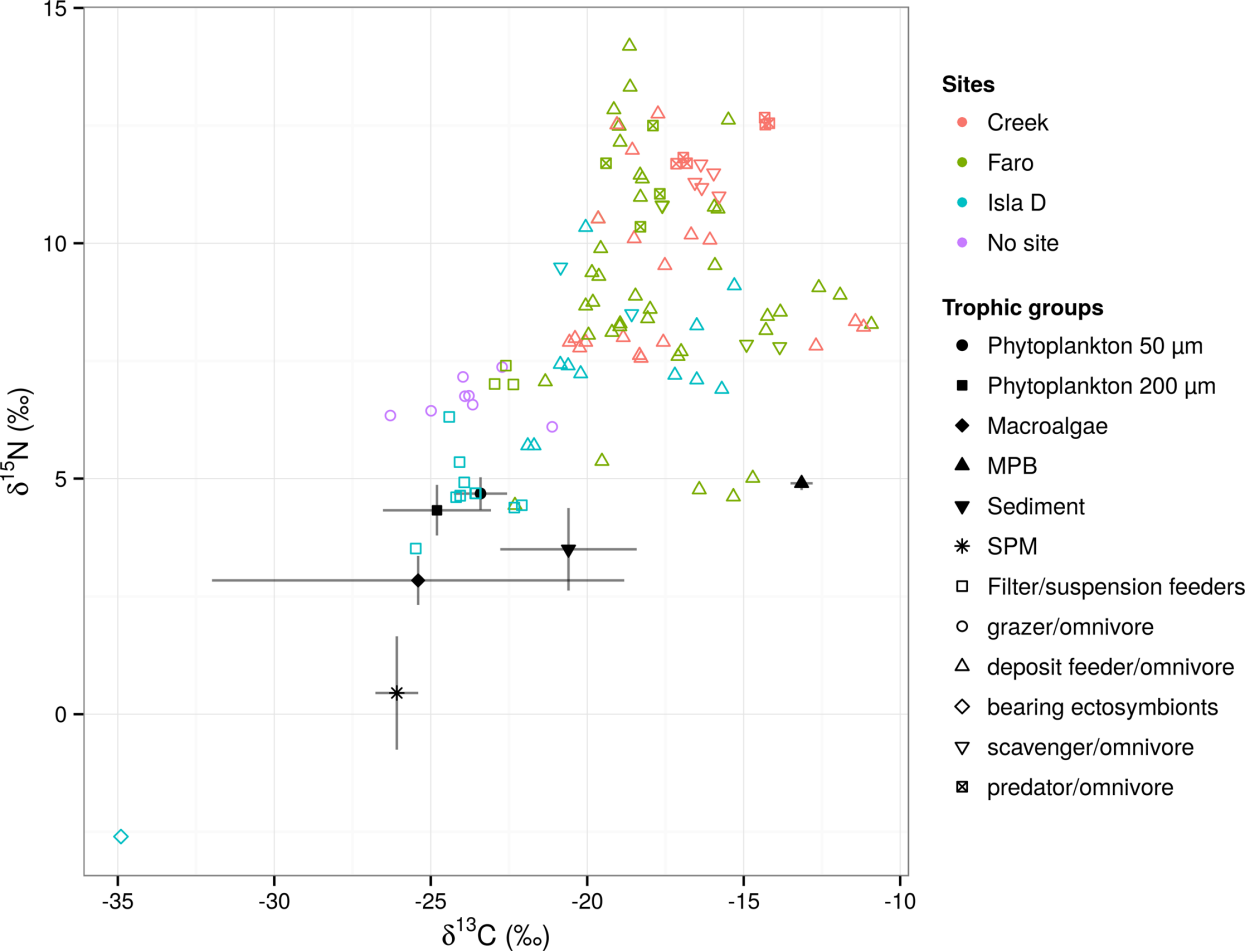
δ^13^C and δ^15^N bi-plot with food sources (filled black symbols) and trophic groups (see legend for symbols) and stations (see legend for colors). The consumers not associated with any site are indicated by purple open circles. “Bearing ectosymbionts” refers to the harpacticoid copepod MT2.

**Fig 3:**
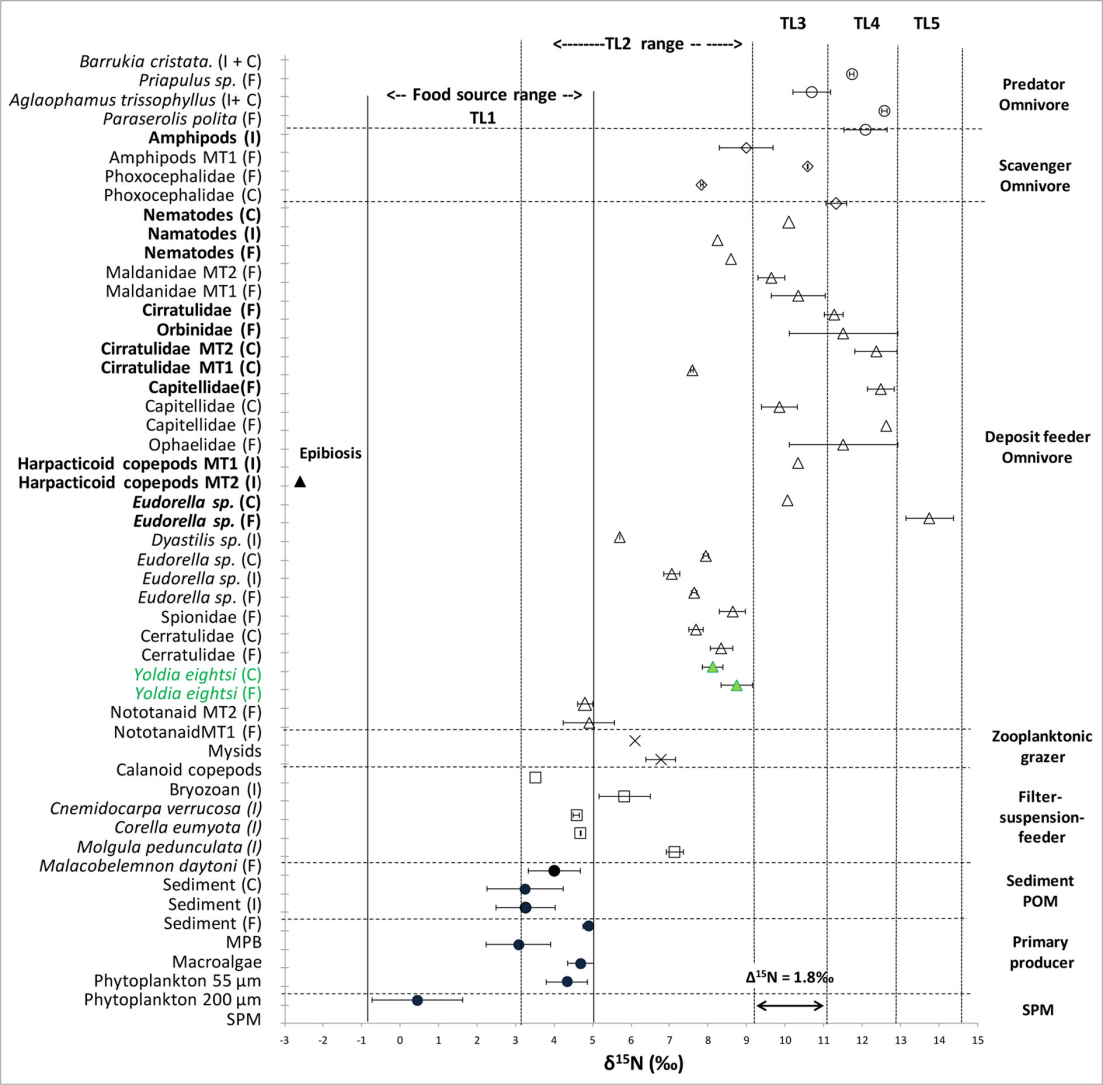
Stable isotope δ^15^N values (food web Δ^15^N = 1.8‰) with taxa trophic group designation. In the list of species/taxa, where appropriate, the site where the organism was sampled is reported in parentheses. Meiofauna taxa are highlighted in bold. In green the baseline organism Yoldia eightsi is highlighted. The abbreviations in listed are Creek = C; Faro = F; Isla D = I; SPM = suspended particulate matter; POM = particulate organic matter; MPB = microphytobenthos; TL = trophic level. Specification of the net mesh size used to sample the phytoplankton is included in the list (55 μm or 200 μm mesh size). Symbols are: filled dark circles = food sources; open squares = filter-suspension feeders; open triangles = deposit feeder/omnivore; open rhombus = scavenger/omnivore; open circles = predator/omnivore.

The isotopic signature (see Fig 2 and S5 Fig) of the food sources ranged from the more depleted carbon isotopic values of macroalgae (mean δ^13^C: -25.03‰ ± 5.45) and SPM (mean δ^13^C: -26.08 ± 1.17 ‰), to the more enriched values of MPB (mean δ^13^C: -13.15‰ ± 0.35). SPM showed overlapping δ^13^C signatures with phytoplankton 200 μm and macroalgae. Macroalgae showed the widest range of δ^13^C values. In terms of δ^15^N signatures SPM showed the most depleted values (0.45‰ ± 1.17) whereas the other food TL ranged around 3–4‰. For the consumers, the deposit feeder/omnivore feeding group showed the widest range of δ^13^C values (-17.82‰ ± 2.57), followed by scavenger/omnivore (-16.76‰ ± 1.79), predator/omnivore (-16.70‰ ± 1.74), zooplankton (-23.80‰ ± 1.41) and filter/suspension feeder (23.51‰ ± 0.95 SD). The most depleted δ^13^C (-34.9‰) as well as δ^15^N (-2.6‰) values were recorded for the harpacticoid copepod MT2 which was the taxon present at Isla D colonized by epibionts (likely ciliates). δ^15^N values of consumers showed a similar pattern with deposit feeder/omnivore holding an intermediate position in the trophic level bi-plot (Fig 3) representing all the consumer trophic levels and showing therefore the widest range of δ^15^N signatures (8.96‰ ± 2.18). The filter/suspension feeder occupied the lower trophic level (TL2, δ^15^N 5.30‰ ± 1.17), whereas scavenger/omnivore (10.17‰ ± 1.14) occupied the range of TL2-TL3 and predator/omnivore (11.86‰ ± 0.71) TL3-TL4 (Fig 3).

### Inter-site comparison: spatial differences in trophic structure

**Fig 4:**
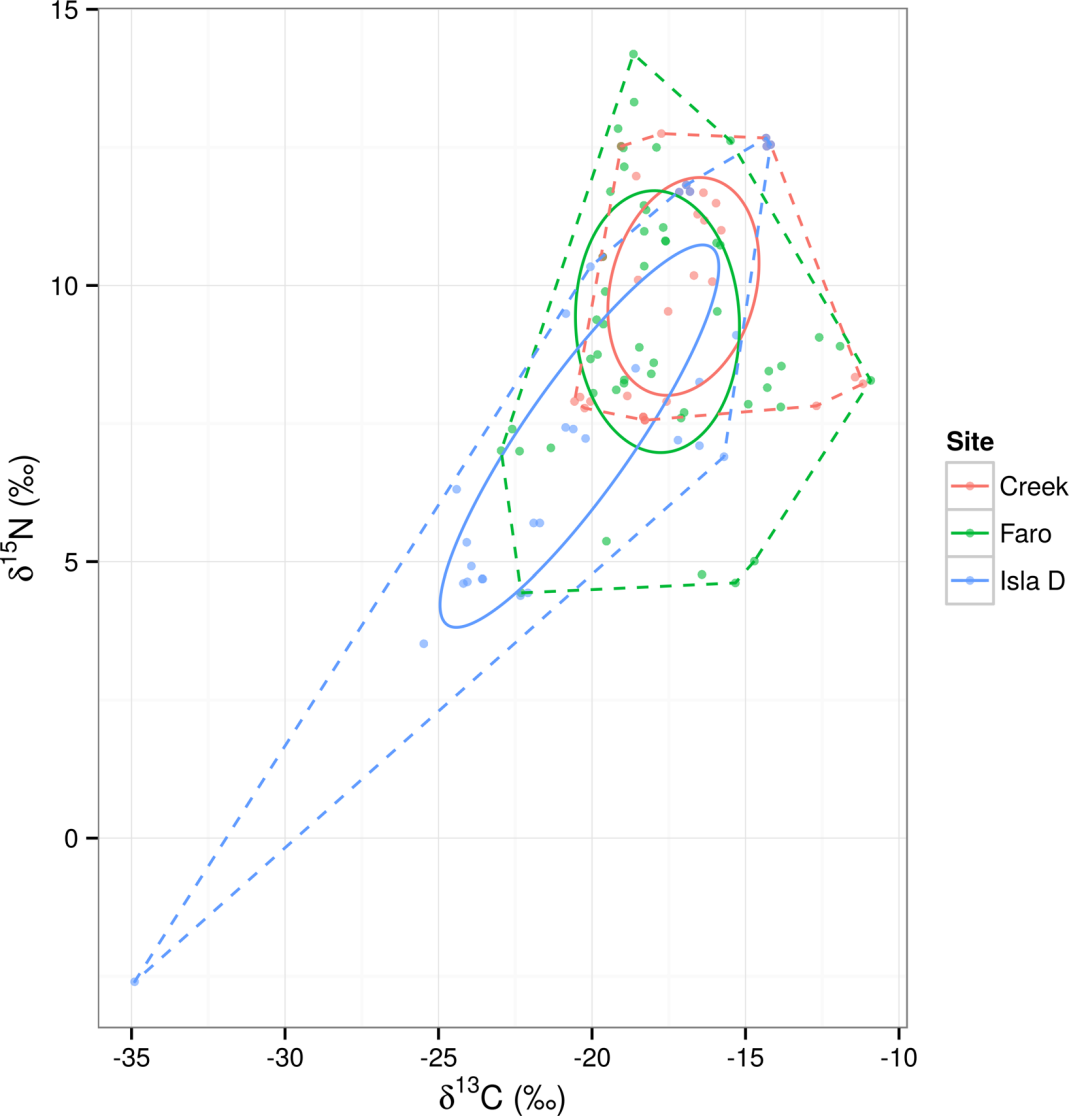
Standard ellipse areas corrected for small samples size. SEA_c_, (full lines) and convex hull areas (dashed lines) for all sites.

**Fig 5:**
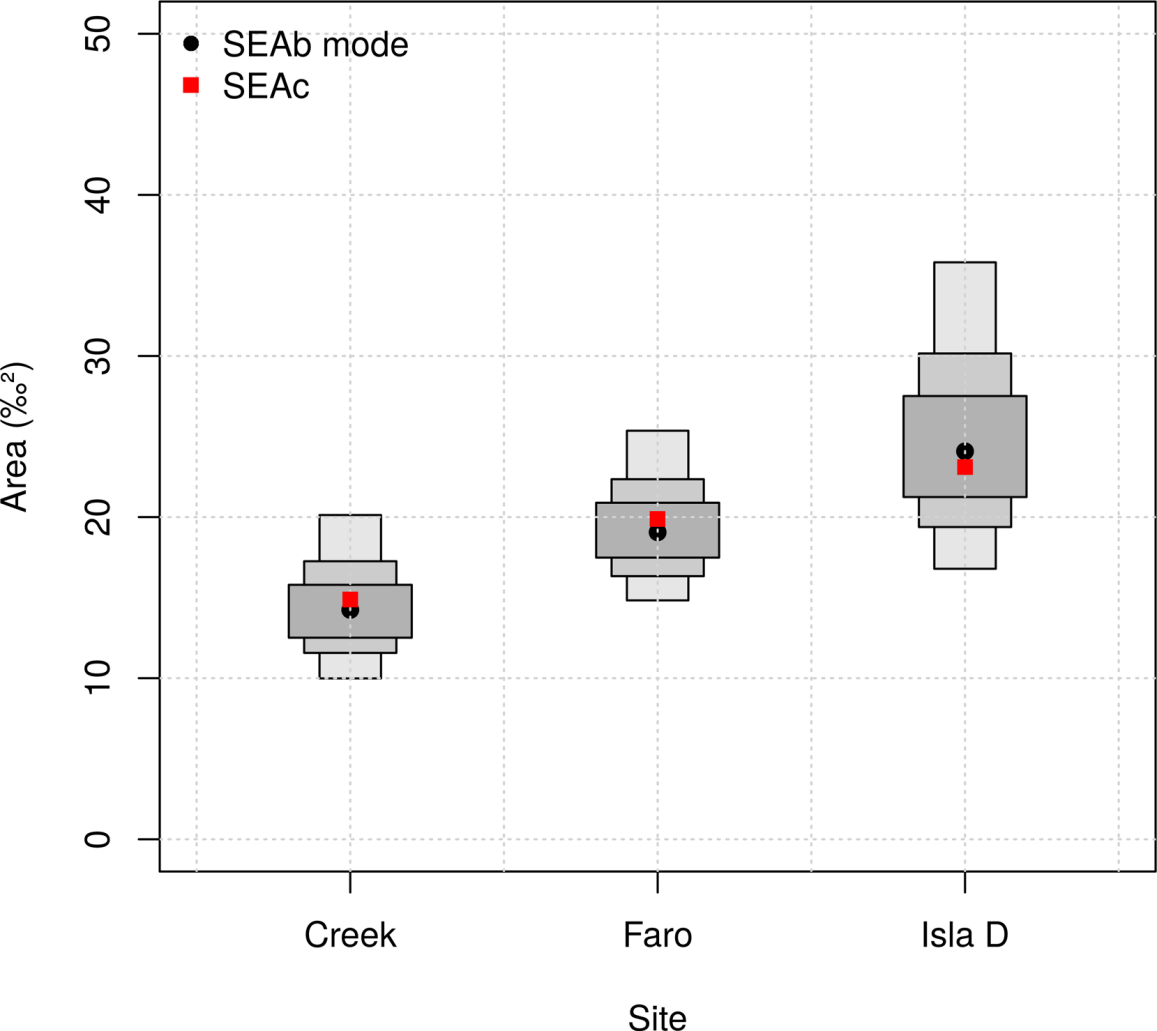
Standard ellipse area Bayesian estimations (SEA_b_). Mode (black dots) and probability of data distribution (50% dark grey boxes; 75% intermediate grey boxes; and 95% light grey boxes) are presented for each site. The standard ellipse area corrected for small sample size (SEA_c_) is also shown as red squares.

**Fig 6:**
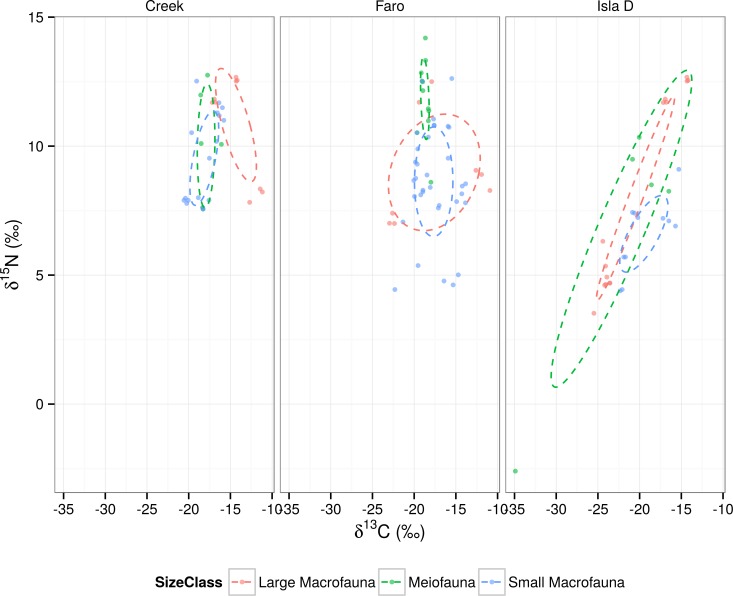
Standard ellipse area corrected for small sample size (SEA_c_). The SEA_c_ are reported for each site with the three consumer size classes (see legend for colors).

Standard ellipse areas corrected for small sample size (SEA_c_) “by site” showed differences in shapes and sizes between the sites (Fig 4). Isla D presented the most elongated and narrow shaped ellipse among the three sites. It covered the largest SEA (23.11‰^2^), followed by Faro (19.88‰^2^) and Creek (14.89‰^2^). The probabilities that the standard ellipse area (SEA_b_) of Isla D was larger than those of Faro and Creek were 0.84 and 0.98, respectively (Fig 5). The probability that the SEA_b_ of Faro was bigger than that of Creek was 0.90. The overlap between Faro and Creek was larger (11.92‰^2^) than between either Creek and Isla D or Faro and Isla D (6.19‰^2^ and 9.15‰^2^, respectively). An SEA_c_ of the “site by size class” (Fig 6, see S2 Table for SIBER results) again showed more elongated and narrow ellipses at the Isla D site. The narrowest ellipses at this site were due to the meiofauna (58.25‰^2^) and “large macrofauna (11.38‰^2^) size classes. Faro displayed a rounder and more compact ellipse in the “large macrofauna size class (37.97‰^2^), whilst the meiofauna and small macrofauna ellipses resembled each other in shape, but differed in size (2.45‰^2^ and 15.37‰^2^, respectively). The three size classes at Creek showed similar SEA_c_ ellipses, both in shape and size (meiofauna 7.92‰^2^; small macrofauna 7.82‰^2^; “large macrofauna 11.65‰^2^), although the overlap could not be compared due to small sample size. The probabilities that meiofauna ellipses were larger than small macrofauna ellipses were 0.38 at Creek, 0 at Faro and 1.00 at Isla D. The probabilities that “large macrofauna ellipses were larger than “small macrofauna ellipses were 0.84 at Creek and 0.99 at Faro and 0.98 at Isla D. In light of the possible “outlier effect” of some specimens (see section “Material and methods”), we decided to repeat the SEAs analysis without including these samples.

**Fig 7:**
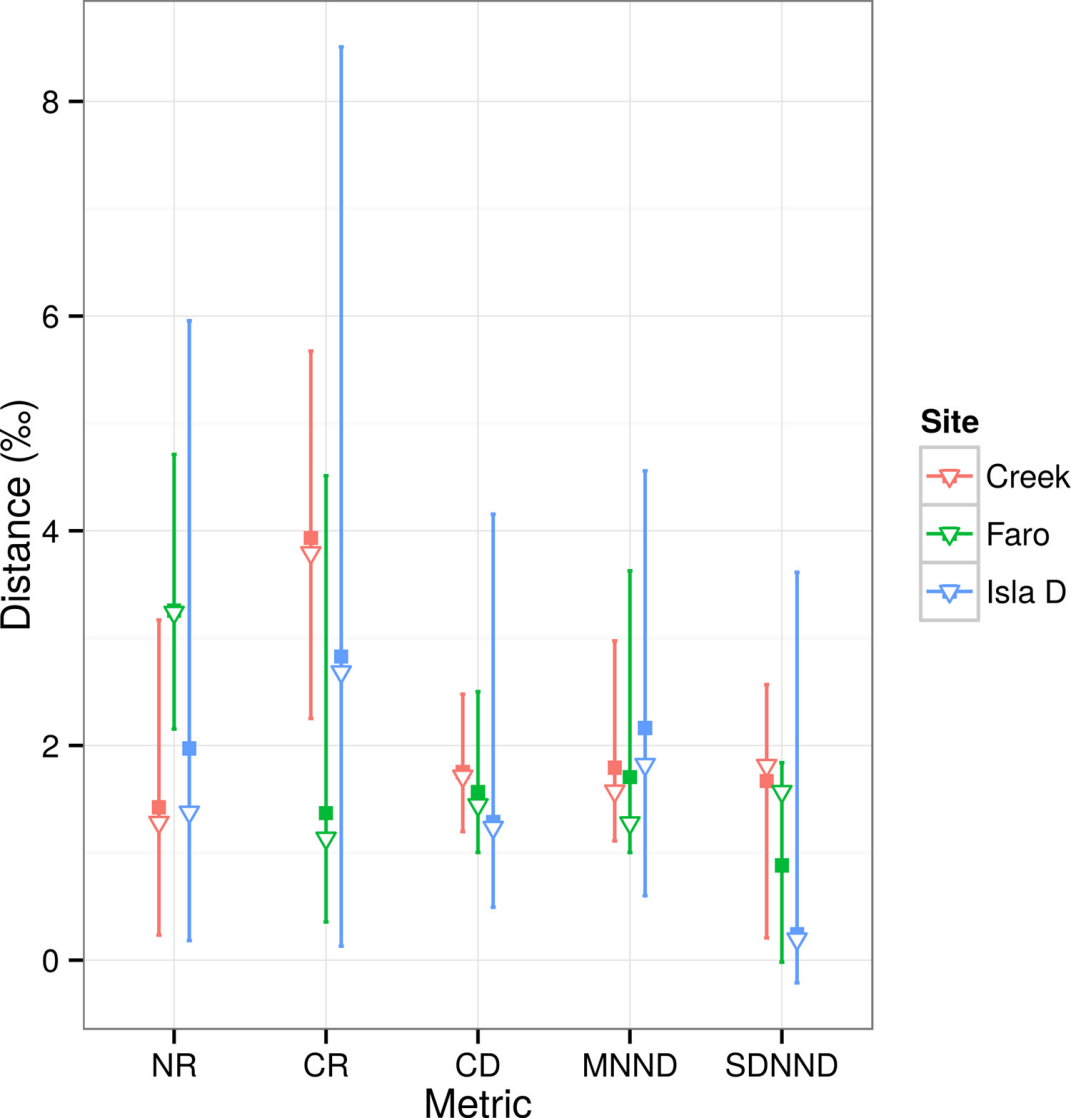
Community-wide metrics. The Layman’s community-wide metrics for the three sites (see legend for colors). The dark squares represent the mode, the triangles the Bayesian probability and the bars the 95% credibility interval of the posterior probability distribution. NR = nitrogen range; CR = carbon range; CD = mean distance from centroid; MNND = mean nearest-neighbor distance; SDMNND = standard deviation MNND.

The Layman’s metrics are summarised in Fig 7 (the Bayesian probability tables are given in S3 Table). In terms of distance (‰), Faro showed the largest NR, with a 0.70 probability of being greater than Isla D and 0.95 probability of being greater than Creek. The CRs of Creek and Isla D were similar (Creek > Isla D = 0.56 probability), whereas Creek and Isla D displayed larger CRs than Faro with 0.90 and 0.76 probability, respectively. Redundancy at the three sites did not show strong differences. The mean distance to centroid (CD) did not show significant differences, with the Credibility Intervals (CI) overlapping between all sites. The mean nearest-neighbour distances (MNND) were relatively similar for the three sites, and did not show significant differences. The CI overlapped between the sites (data not shown).

When running the analysis without the harpacticoid copepod MT2 and the two polychaete species in Isla D, we found an important influence of these samples on the overall *inter-site comparison* results (see S1–S3 Tables for summary). The *a posteriori* Bayesian probability pointed now to an inverted order in the SEA_s_, with Faro showing the larger area, followed by Creek and Isla D, with nevertheless relatively small differences. Excluding the epibiosis life strategy from the analysis and hence from the meiofauna size class, the *site by size comparison* (see S3 Fig) analysis showed significant differences from the “*complete dataset”* analysis, with increasing trophic niche width at increasing size spectra (large macrofauna > small macrofauna > meiofauna). At Isla D the large macrofauna SEA_c_ now showed very reduced trophic niches, and the meiobenthos occupied the highest position compared to the other two size classes (S3 Fig).

## Discussion

### Temporal and spatial glacier retreat effects on benthic trophic interactions

#### Spatial patterns: isotopic niche width

The degree of compaction of the three isotopic niches studied showed differences in line with the ice-free age status of each site, and the associated environmental conditions at the time of sampling. The most recently ice-free station (Isla D) hosted the least compact and widest isotopic niche, whereas the site known to be ice-free for the longest time (Creek) had the most compacted SEA_c_ shape, indicating higher efficiency in terms of biomass transfer efficiency theory [93].

This trend indicates the potential ability of the Antarctic benthic assemblage to increase the degree of interconnectedness within the food chain during a relatively short (years to decades) time after an important environmental change (in this case the newly ice-free status). This would suggest these communities have a good capacity to adapt to the strong ongoing climate-driven changes.

The very elongated shape of the SEA_c_ ellipse at Isla D was mainly due to the meiofauna size class assemblage. Within this size group, the epibiosis trophic strategy identified in the meiobenthic harpacticoid copepod (MT2) was present only at this site. This morphotype stable isotope analysis showed negative δ^15^N and very depleted δ^13^C values, indicative of symbiosis and chemosynthetic relationships. At Isla D, cumaceans were also found to carry epibiont ciliates on their exoskeleton (F. Pasotti, *pers*. *observ*.). This strategy was not observed in the other two sites during our sampling. In our earlier study we identified the relatively greater dominance of the pioneer coloniser nematode genus *Microlaimus* and the higher abundance of harpacticoid copepods at the site [44]. *Microlaimus* is known to be a successful colonizer of fresh ice-scoured sediments (94) and to be present in intermediate/late stages of succession after the collapse of the Larsen B ice shelf [39]. Similarly, harpacticoid copepods have been reported to be rapid colonisers of scoured [94] and artificial bare [95] sediments. With their specific functional traits, meiobenthic organisms can respond to high levels of ice disturbance and the newly available resource pool via rapid colonization processes, then establishing more differentiated trophic niches during the early years after these events.

Isla D is the site which remained under the glacier tongue for the longest time. The local benthic assemblage here may have undergone a successional process that resulted in species impoverishment. This is observed on islands where the communities typically show an expanded niche width compared to their mainland counterparts [96]. Terrestrial examples of succession following glacier retreat in the Antarctic showed a general increase in diversity of plants, mites and nematodes with time since the habitat became deglaciated [15,97,98]. At both Faro and Isla D, as a consequence of ice retreat, new substrate became available for colonization by macroalgae [14]. Further, we recently observed that at Isla D there was a high occurrence (~40% of surface sediment microalgae) of sea-ice and phytoplanktonic algae [44]. We observed that both indicators of isotopic niche width (SEA_c_ and SEA_b_) were larger for Isla D and to a lesser extent also in Faro, compared to Creek. The additional resource pool represented by these primary producers could provide an explanation for the wider isotopic niche (wider primary producer isotopic niche) of the local assemblages found at these two sites.

Strong benthic-pelagic coupling was suggested to sustain a detritus-based sediment community at Faro site [44] where macrofauna remineralised detritus (e.g. faecal pellets) becomes available as reworked food for the meiobenthic deposit feeders. Nevertheless our data (see generally intermediate δ^13^C values of benthic consumers, e.g. deposit feeder/omnivore group, and values in Table 1) indicate that MPB were an important food source for the benthic compartment and the meiofauna. At Faro, the SEA_c_ showed an intermediate position and a compacted shape, with relatively higher trophic levels (δ^15^N values) of meiofauna taxa. This may indicate that both the macrobenthos and the meiobenthos rely on a mixture of food sources (fresh microphytobenthic production and detrital matter) and meiofauna organisms may include organisms with likely predatory feeding habits in their diets.

From the present data, the isotopic niche width at Creek was the smallest and showed the highest relative position in the bi-plot space. The older ice-free age, the high degree of disturbance resulting from the adjacent glacial meltwater stream and the likely frequent re-suspension and ice-scouring events characteristic for the location, may force the community to a more efficient use of the available resources which are finally transferred to the higher trophic levels. Not surprisingly, the SEA_c_ ellipse of the Creek community overlapped by 80% with that of Faro, suggesting a similar “centralized” isotopic niche with a short food chain and efficient resource use.

#### Spatial patterns: redundancy

When species with different life strategies colonize a ‘new’ environment the functional diversity increases locally, and so the trophic niche of the community is expected to widen. With time an increase in trophic redundancy can be expected, since newcomers often share similar functional traits, as already observed for stream communities after glacial retreat in south-east Alaska [99]. Our analyses showed that the three assemblages considered here did not display significant differences in trophic redundancy, although MNND was always slightly higher at Isla D compared to the two other stations (i.e. lower species packing or lower trophic redundancy) and Creek showed a slightly higher SDMNND than the other two sites (i.e. less even distribution of trophic niches or lower trophic redundancy). Isla D and Creek are the sites that experience higher levels of ice disturbance and which showed the most patchiness in the community distribution [44]. However, the Bayesian probabilities for these differences can be questioned (they were never higher than 0.59, see S3 Table) and a lack of differences in redundancy seems the most plausible explanation. The sites had already been ice-free for several years at the time of sampling (at least eight years for Isla D) and, because of their small distance and the local cyclonic current system [52,53], the communities may have already shared most of the species/taxa (and hence, most of the trophic diversity). Finally, the very high degree of omnivory that appears to characterize the Potter Cove benthos trophic network (see section “Temporal and spatial glacier retreat effects on benthic trophic interactions”) and the fact that our observations cannot be considered indicative of a primary colonization, may hamper the utility of these metrics to detect the colonization direction in this shallow water environment.

#### Size by site comparison: trophic niche width

A “size by site” comparison of the SEA_c_ ellipses (Fig 6) showed specific patterns for the different sites. The meiofauna ellipse was wider at Isla D and it encompassed the isotopic niche ellipses of the two macrofauna size classes. At Isla D site a continuous intermediate level of ice-scour disturbance exists and re-colonisation can be locally stimulated. Most of the macrofauna biomass was made up by mobile scavengers and predators and, overall, was relatively low compared to the other two sites [50], whereas the meiofauna were numerous and comparable in biomass to the other study locations. Meiofauna taxa are known to be rapid re-colonizers after ice-scour [94] and to be less sensitive to sediment mechanical instability than macrofauna [100].

At Faro the SEA_c_ of meiobenthic organisms is positioned above the two macrobenthos ellipses. This may indicate that the meiofauna rely on macrobenthic-derived detritus for their diet. Other authors [70,78] have also suggested that the faecal pellets of filter (and suspension) feeders could be an important source of organic matter for the surrounding sediments.

Creek benthos did not show any clear pattern in relation to the size class ellipses. The food chain appeared more compact, and the recycling of the locally available organic matter is likely to be efficient, possibly linked to the high level of sediment re-suspension and remixing. Furthermore, the food web appears to be dominated by deposit feeders, scavengers and predators, as seen at Isla D, likely to result from high levels of ice-scour and related mortality. This high level of ice-scour is likely to generate large amounts of dead animal material on the sediment, and the recycling and reutilization of this organic matter may underlie the high position of the ellipses in the bi-plot space.

### Reduced dataset analysis: highlights

Isla D large macrofauna appeared to exploit mostly the basal resources (lower δ^15^N), lacking the higher TL_s_. Further, the size spectra ellipses (or isotopic niches, see S3 Fig) show a clear separation at the most recently ice-free site compared to those of Creek and Faro. The removal of the copepod MT2 and of the higher TL_s_ of the large macrofauna from the Isla D dataset resulted in more centered ellipses with a lower dispersion of the data. This was statistically confirmed by the observation of the Layman’s metrics and related Bayesian statistic for the three sites comparison (see S3 Table). After their removal, Isla D showed larger values for all the metrics compared to Creek and Faro, with a minimum Bayesian posterior probability of > 0.80. This outcome is plausible given that, by reducing the simulated ranges at Isla D (by taking away the “outliers”), the mean became higher. Interestingly, from the observation of the MNND metric (a measure related to the overall density of species packing,[74]), a significantly higher value was obtained after removal of these outliers—and hence indicating lower trophic redundancy—at Isla D. By removing only a few life strategies in the analysis, the new results highlighted the importance of the “time-since-ice-retreat” in the overall interpretation of the metrics and SEA_s_; the less time a site had been available for colonization (Isla D), the lower the redundancy in trophic functions found within its community [101]. Finally in this “*reduced dataset”* analysis, higher trophic diversity (SEA_s_) was apparent where the level of disturbance (e.g. ice scouring and inorganic sedimentation) was intermediate to low (Faro), a pattern already observed elsewhere in streams [102]. The two analyses complement each other in providing a wider view of the potential underlying processes that shape the shallow benthic community assemblages at these inner sites in Potter Cove.

### General considerations: functional traits in Potter Cove benthic food web

Overall, the benthic food web at the three Potter Cove contrasting sites showed the presence of several levels of consumers spanning from the wide stable isotopic range of deposit feeder/omnivore to the more defined range of predator/omnivore and scavenger/omnivore. We identified a total of four consumer trophic levels (TL_s_) by means of a reduced (Δδ^15^N = 1.8‰) invertebrate/plant based-diet fractionation step [64]. The studied benthic organisms mostly feed on other invertebrates (e.g. by scavenging, predation or unspecific ingestion of sediment) or on various types of detritus (e.g. by selective or non-selective deposit feeding) which may be of both algal (fresh or dead micro-/macroalgae) or invertebrate-derived (e.g. faecal pellets, reworked invertebrate carcasses) origin. This generalist feeding behavior adds more levels of recycling within the food web, generating a “trophic continuum”. This could eventually hide (isotopically) the real trophic role an organism could effectively cover (e.g. many polychaetes can be predators on smaller invertebrates but also feed unselectively on microalgae), thereby shortening the total food chain length. This trophic continuum can be also reflected in the width of the stable isotopic signature of the deposit feeder/omnivore consumer group and in the overlap of the various consumer categories, highlighting how difficult it is to segregate the trophic categories based on their nitrogen isotopic signatures. Overall there may be more feeding plasticity than estimated, and trophic fractionation needs to be fitted to the actual diet of the organisms.

The food web in Potter Cove has long been suggested to very likely rely on macroalgal detritus [103] in view of the locally high benthic biomasses and the usually low local phytoplankton production [104] and the presence of seaweed detritus in filter and suspension feeder key species in Potter Cove [70,105]. Moreover many grazing amphipods can feed directly on this algal resource pool. Another potential algal food source, the microphytobenthos (MPB), has not yet been quantified in the cove, but its importance as food for the benthos has been suggested in other studies for the adjacent Admiralty Bay benthos [48]. In this investigation macroalgae presented a wide range of δ^13^C values, overlapping with the sediment carbon signatures, but with a rather depleted signal. The majority of the organisms examined displayed intermediate to enriched δ^13^C values, likely pointing to the importance of fresh summer microphytobenthos for the Cove food web. The MPB signal was the most enriched of the studied food sources. For instance, the baseline organism *Yoldia eightsi* showed a rather clear MPB signal, since its carbon values were slightly more enriched (by 1.35‰) than those of the microalgae. Considering the most important meiobenthic group, the nematodes, a strong link is apparent to the local signature of sediment (Table 1 and S5 Fig). Nematode assemblages in Potter Cove are generally dominated by non-selective deposit feeding taxa [44,106], and the intermediate value we found in this study lies between their site-specific sediment value and the MPB value, confirming some feeding plasticity at the community level. Finally, the very depleted δ^15^N values of some filter feeder species may point to a higher contribution of SPM to the diet of these organisms rather than phytoplankton. The SPM also helps to explain the large width we identified for the first consumer trophic level (TL1) when considering the water column and sediment as one benthic-pelagic trophic environment. Nevertheless, it is important to stress that the sampling protocol for phytoplankton collection used in this study may not have sampled those components smaller than 55 μm (the lower limit of our mesh size) and hence failed to find possible trophic connections between smaller phytoplankton and the filter feeders investigated.

Bacterial degradation may affect carbon and nitrogen stable isotope organic matter signatures [107], often significantly increasing or decreasing the δ^15^N values. Organic matter recycling may have an important role in the Cove’s sediments due to the abundant bacterial community [44]. The tighter connection that the meiobenthos has with the “small food web” of microrganisms may be one of the reasons behind the generally high δ^15^N values of these small metazoan taxa (see Figs 3 and 6). Reworked organic matter and other type of detritus (e.g. filter feeders’ faecal pellets) can enter the higher trophic levels of the benthic system via deposit feeding, or else be available to benthic suspension feeders via the frequent re-suspension events during the summer months. Taking this into account, omnivory (feeding plasticity with potential to feed on microalgae, macroalgae and bacteria) and deposit feeding (feeding on unselected sediment organic matter) appear to be effective strategies in this shallow water polar system.

## Supporting Information

S1 Fig Standard ellipse areas corrected for small samples size (SEA_c_). SEA_c_, full lines) and convex hull area (dashed lines) for all sites (see legend) for the “*reduced dataset”* analysis.
(TIF)

S2 Fig Standard ellipse area Bayesian estimations (SEA_b_) for the “*reduced dataset”* analysis. Mode (black dots) and probability of data distribution (50% dark grey boxes; 75% intermediate grey boxes; and 95% light grey boxes) for each site are presented. The standard ellipse area corrected for small sample size (SEA_c_) is also presented as red squares.
(TIF)

S3 Fig Standard ellipse area corrected for small sample size (SEA_c_) for the “reduced dataset” analysis. For each site the three consumer size classes (see legend for colors) are represented.
(TIF)

S4 Fig Community-wide metrics for the three sites. The dark squares are the mode, the triangles the Bayesian probability and the bars represent the 95% credibility interval of the posterior probability distribution. NR = nitrogen range; CR = carbon range; CD = mean distance from centroid; MNND = mean nearest-neighbor distance; SDMNND = standard deviation MNND. See legend for colors.
(TIF)

S5 Fig Stable isotope δ^13^C values of food sources and consumers. In the list of species/taxa, where appropriate, the site where the organism was sampled is reported in parentheses. Meiofauna taxa have been highlighted in bold. The baseline organism *Yoldia eightsi* is highlighted in green. The abbreviations in the list are Creek = C; Faro = F; Isla D = I; SPM = suspended particulate matter; POM = particulate organic matter; MPB = microphytobenthos; TL = trophic level. Specification of the net mesh size used to sample the phytoplankton is included in the list (55 μm or 200 μm mesh size). Symbols refer to trophic group designation as from Fig 3 in the manuscript.
(TIF)

S1 Table SIBER analysis comparison “by site”. Comparison of SIBER analysis results for the "*complete dataset*” analysis (left) and the "*reduced dataset*” analysis (right) for the “*by site*” analysis.
(DOCX)

S2 Table SIBER analysis comparison “site by size”. Comparison of SIBER analysis results for the "*complete dataset*’ analysis (left) and the "*reduced dataset*” analysis (right) for the “*size by site*” analysis.
(DOCX)

S3 Table Community-wide metrics pairwise comparison. Pairwise comparison for each site using Bayesian posterior probabilities [73]. Results are shown for both analysed datasets.
(DOCX)
