# Supplementary figures and images for: Benthic Trophic Interactions in an Antarctic Shallow Water Ecosystem Affected by Recent Glacier Retreat
Source: PLoS One. 2015 Nov 11;10(11):e0141742. doi: 10.1371/journal.pone.0141742 (PMC4641631; doi:10.1371/journal.pone.0141742)

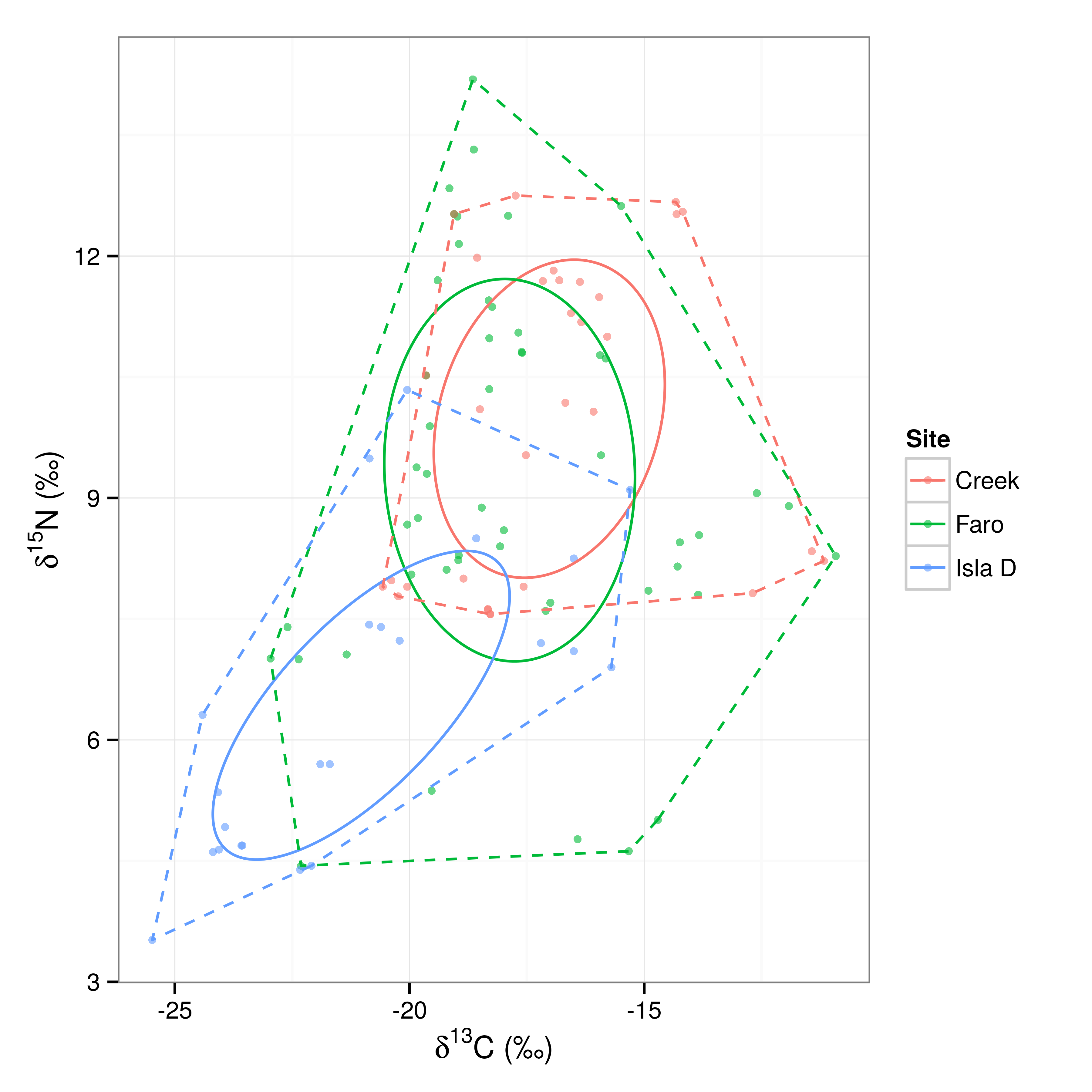

Supplement: S1 Fig — SEAc, full lines) and convex hull area (dashed lines) for all sites (see legend) for the “reduced dataset” analysis. (TIF) [file pone.0141742.s001.tif]

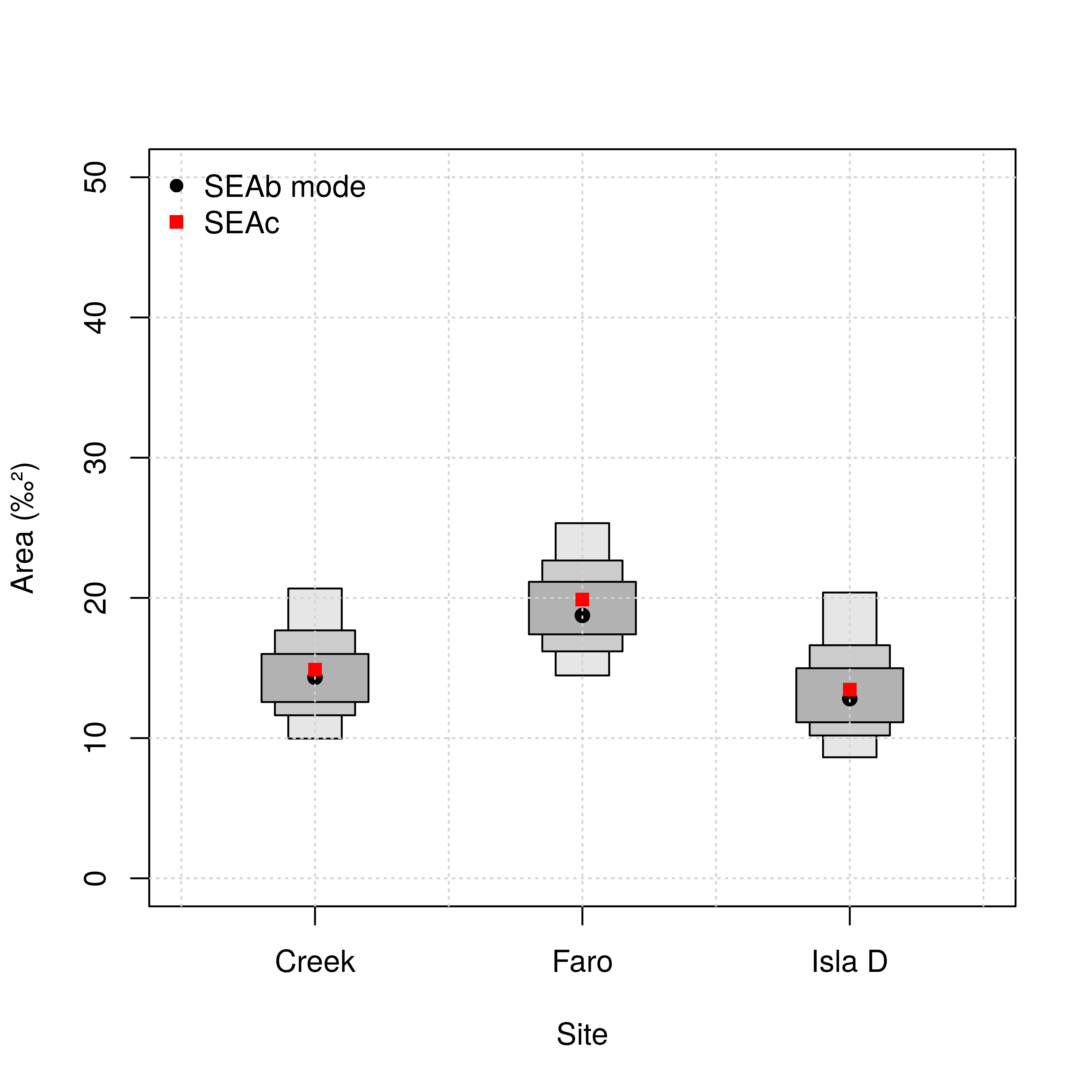

Supplement: S2 Fig — Mode (black dots) and probability of data distribution (50% dark grey boxes; 75% intermediate grey boxes; and 95% light grey boxes) for each site are presented. The standard ellipse area corrected for small sample size (SEAc) is also presented as red squares. (TIF) [file pone.0141742.s002.tif]

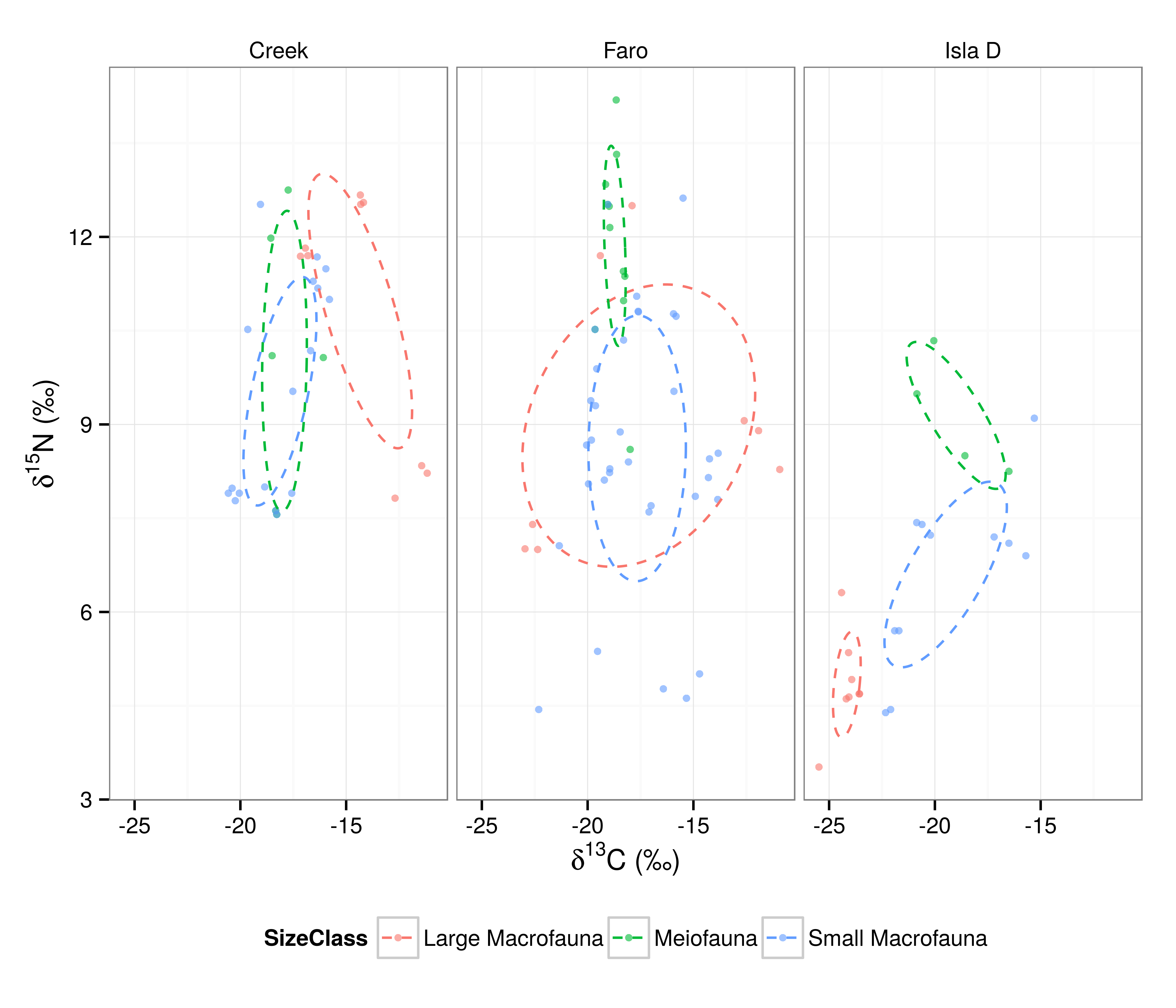

Supplement: S3 Fig — For each site the three consumer size classes (see legend for colors) are represented. (TIF) [file pone.0141742.s003.tif]

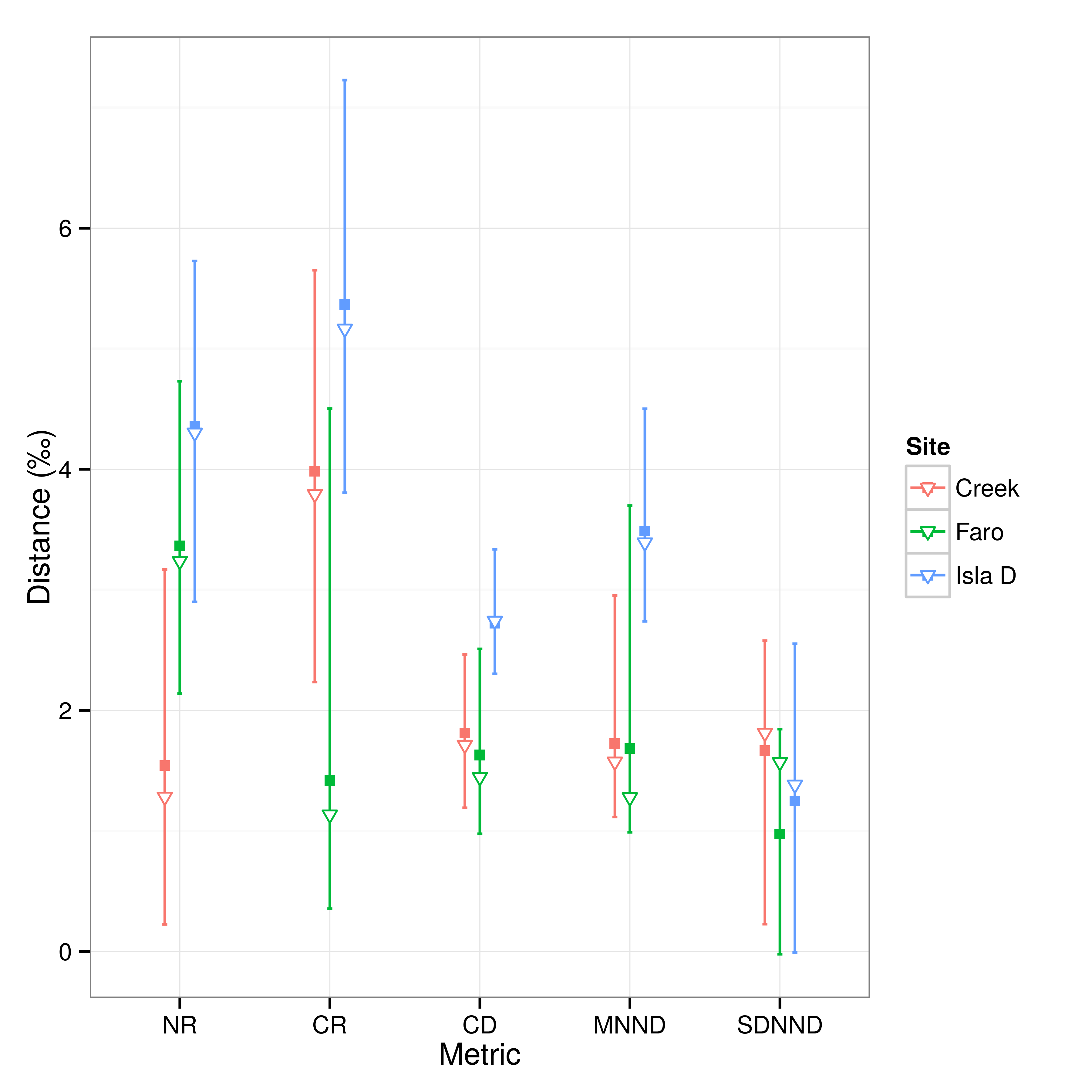

Supplement: S4 Fig — The dark squares are the mode, the triangles the Bayesian probability and the bars represent the 95% credibility interval of the posterior probability distribution. NR = nitrogen range; CR = carbon range; CD = mean distance from centroid; MNND = mean nearest-neighbor distance; SDMNND = standard deviation MNND. See legend for colors. (TIF) [file pone.0141742.s004.tif]

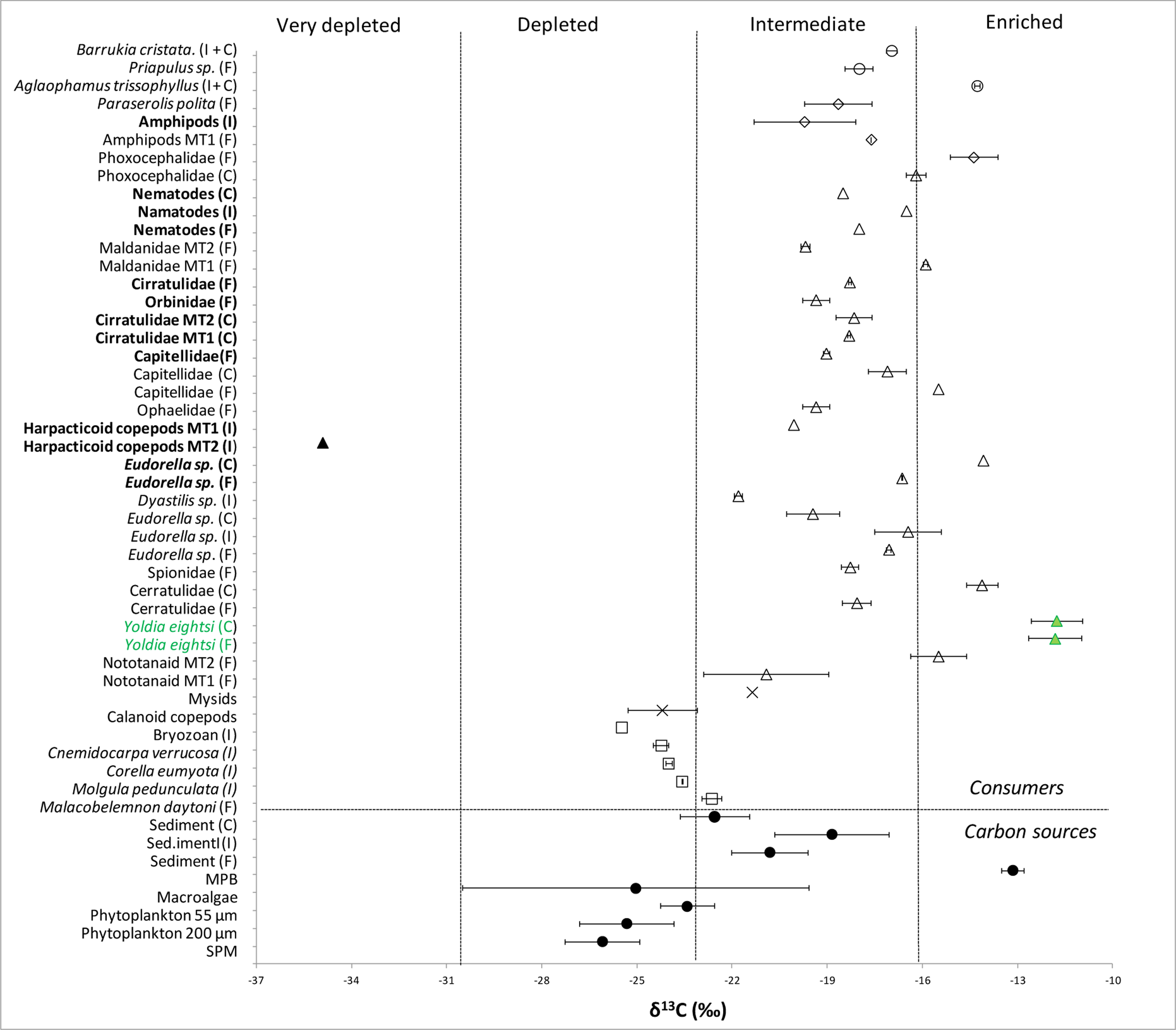

Supplement: S5 Fig — In the list of species/taxa, where appropriate, the site where the organism was sampled is reported in parentheses. Meiofauna taxa have been highlighted in bold. The baseline organism Yoldia eightsi is highlighted in green. The abbreviations in the list are Creek = C; Faro = F; Isla D = I; SPM = suspended particulate matter; POM = particulate organic matter; MPB = microphytobenthos; TL = trophic level. Specification of the net mesh size used to sample the phytoplankton is included in the list (55 μm or 200 μm mesh size). Symbols refer to trophic group designation as from Fig 3 in the manuscript. (TIF) [file pone.0141742.s005.tif]
