# Supplementary material for: Benthic Trophic Interactions in an Antarctic Shallow Water Ecosystem Affected by Recent Glacier Retreat
Source: PLoS One. 2015 Nov 11;10(11):e0141742. doi: 10.1371/journal.pone.0141742 (PMC4641631; doi:10.1371/journal.pone.0141742)
Supplement: S1 Table — Comparison of SIBER analysis results for the "complete dataset” analysis (left) and the "reduced dataset” analysis (right) for the “by site” analysis. (DOCX) [file pone.0141742.s006.docx]

**S1 Table. Comparison of the SIBER analyses “by site”.**

|  | ***By site analysis*** | |  |
| --- | --- | --- | --- |
|  | ***complete dataset*** | ***reduced dataset*** |  |
| SEA_c_ area (‰^2^) | | | |
| Faro | 19.88 | 19.88 |  |
| Isla D | 23.11 | 13.47 |  |
| Creek | 14.89 | 14.89 |  |
| Bayesian posterior probabilities for SEA_b_ by site | | | |
| Faro > Creek | 0.90 | 0.90 |  |
| Isla D > Creek | 0.98 | 0.40 |  |
| Isla D > Faro | 0.84 | 0.09 |  |
| SEA_c_ Overlaps by site (‰^2^) | | | |
| Faro and Creek | 11.92 | 11.92 |  |
| Isla D and Creek | 6.19 | 0.0968 |  |
| Isla D and Faro | 9.15 | 1.96 |  |
