# Supplementary material for: Benthic Trophic Interactions in an Antarctic Shallow Water Ecosystem Affected by Recent Glacier Retreat
Source: PLoS One. 2015 Nov 11;10(11):e0141742. doi: 10.1371/journal.pone.0141742 (PMC4641631; doi:10.1371/journal.pone.0141742)
Supplement: S2 Table — Comparison of SIBER analysis results for the "complete dataset’ analysis (left) and the "reduced dataset” analysis (right) for the “size by site” analysis. (DOCX) [file pone.0141742.s007.docx]

**S2 Table. Comparison of the SIBER analyses “size by site”.**

|  | ***Size by site analysis*** | | | | | |
| --- | --- | --- | --- | --- | --- | --- |
|  | ***complete dataset*** | | | ***reduced dataset*** | | |
|  | SEA_c_ area (‰^2^) | | | | | |
|  | *Faro* | *Isla D* | *Creek* | *Faro* | *Isla D* | *Creek* |
| meiofauna | 2.45 | 58.25 | 7.92 | 2.45 | 5.18 | 7.92 |
| small macrofauna | 15.38 | 9.28 | 7.82 | 15.38 | 9.28 | 7.82 |
| large macrofauna | 37.97 | 11.38 | 11.65 | 37.97 | 1.62 | 11.65 |
|  | Bayesian posterior probabilities for SEA_b_ by site | | | | | |
|  | *Faro* | *Isla D* | *Creek* | *Faro* | *Isla D* | *Creek* |
| meiofauna > small macrofauna | 0 | 1.00 | 0.38 | 0 | 0.16 | 0.40 |
| large macrofauna > small macrofauna | 0.99 | 0.98 | 0.84 | 0.99 | 0 | 0.83 |
| large macrofauna > meiofauna | 1.00 | 0.01 | 0.85 | 1.00 | 0.02 | 0.84 |
