# Supplementary material for: Benthic Trophic Interactions in an Antarctic Shallow Water Ecosystem Affected by Recent Glacier Retreat
Source: PLoS One. 2015 Nov 11;10(11):e0141742. doi: 10.1371/journal.pone.0141742 (PMC4641631; doi:10.1371/journal.pone.0141742)
Supplement: S3 Table — Pairwise comparison for each site using Bayesian posterior probabilities [73]. Results are shown for both analysed datasets. (DOCX) [file pone.0141742.s008.docx]

**S3 Table. Comparison of the Layman’s metrics.**

|  |  | ***complete dataset*** | |  |  |
| --- | --- | --- | --- | --- | --- |
|  | dNR | dCR | CD | MNND | SDNND |
| Creek > Faro | 0.0433 | 0.9009 | 0.6181 | 0.4394 | 0.7622 |
| Creek > Isla D | 0.2704 | 0.5662 | 0.5184 | 0.3555 | 0.704 |
| Faro > Isla D | 0.7017 | 0.2384 | 0.4614 | 0.4169 | 0.5192 |
|  |  | ***reduced dataset*** | |  |  |
| Creek > Faro | 0.046 | 0.9005 | 0.6329 | 0.45 | 0.7637 |
| Creek > Isla D | 0.01 | 0.1045 | 0.0138 | 0.0123 | 0.5956 |
| Faro > Isla D | 0.17 | 0.0208 | 0.0209 | 0.0598 | 0.3408 |
